# Experimental Investigation Table-moving

**Published:** 1853-08

**Authors:** 


### EXPERIMENTAL INVESTIGATION OF TABLE-MOVING.

Our readers cannot be unaware of the excitement which prevails, not only in this country, but in London and Paris, on the subject of certain phenomena styled "table-turning," "table-tipping," &c., and which have been erroneously referred to electricity, magnetism, a spiritual, or diabolical agency, or some new force in nature not yet understood. The evil of it has already been very great, for as most people adopt the supernatural theory of the matter a morbid feeling has been created which, in not a few instances, has terminated in insanity. Prof. Faraday, one of the most eminent philosophers of our times, has engaged in an experimental investigation of the subject, and the results of his inquiry have been published in the *London Athenaeum*. They are so satisfactory, so perfectly conclusive, that we wish to give as wide a circulation to them as possible. Prof. F. prefaces the account of his experiments by saying—

"I have been greatly startled by the revelation which this purely physical subject has made of the condition of the public mind. No doubt, there are many persons who have formed a right judgment or used a cautious reserve—for I know several such, and public communications have shown it to be so; but their number is almost as nothing to the great body who have believed and borne testimony, as I think, in the cause of error. I do not here refer to the distinction of those who agree with me and those who differ. By the great body I mean such as reject all consideration of the equality of cause and effect—who refer the results to electricity and magnetism, yet know nothing of the laws of these forces—or to attraction, yet show no phenomena of pure attractive power—or to the rotation of the earth, as if the earth revolved round the leg of a table; or to some unrecognized physical force, without inquiring whether the known forces are not sufficient—or who even refer them to diabolical or supernatural agency, rather than suspend their judgment, or acknowledge to themselves that they are not learned enough in these matters to decide on the nature of the action. I think the system of education that could leave the mental condition of the public body in the state in which this subject has found it must have been greatly deficient in some very important principle."

The object which Prof. F. had in view in this inquiry, he says, was not to satisfy himself, for he was convinced as to the true explanation of the phenomena by the reports of the table-turners themselves, "but that he might be prepared to give a strong opinion, founded on facts," to the many who applied to him for it. The methods which he adopted were precisely such as he would have followed in any other physical investigation. The parties with whom he worked were very honorable, very clear in their intentions, successful table-movers, very desirous of succeeding in establishing the existence of a pecular power, thoroughly candid, and very effectual." With him it was "a clear point that the table moves when the parties, though they strongly wish it, do not intend, and do not believe that they move it by ordinary mechanical power." The account they give is, that "the table draws their hands; that it moves first and they have to follow it; that sometimes it moves from under their hands." "Though," says Prof. F. "I believe the parties do not intend to move the table, but obtain the result by a *quasi* involuntary action, still I had no doubt of the influence of expectation upon their minds and through that upon the success or failure of their efforts." He proceeded to test this opinion, and so arranged his apparatus as to show any electricty that might be evolved, and at the same time any pressure that might be exerted by the table-mover. Not the slightest trace of electrical or magnetic effects was obtained," and no experiment "gave the slightest indication of any peculiar natural force. He adds: "

"No attractions, or repulsions, or signs of tangential power appeared —nor anything which could be referred to other than the mere mechanical pressure exerted inadvertently by the turner. I therefore proceeded to analyze this pressure, or that part of it exerted in a horizontal direction, doing so, in the first instance unawares to the party."

We omit his description of his apparatus, simply remarking that the arrangement was such as to be perfectly convincing to the table-mover himself whether or not he was using pressure. The result was that,

"On examination, it was easy to see by the displacement of the parts of the line, that the hand had moved further than the table, and that the latter had lagged behind—that the hand, in fact, had pushed the upper card to the left, and that the under cards and the table had followed and been dragged by it. In other similar cases when the table had not moved, still the upper card was found to have moved, showing that the hand had carried it in the expected direction. It was evident, therefore, that the table had not drawn the hand and person round, nor had it moved simultaneously with the hand. The hand had left all things under it behind, and the table evidently tended continually to keep the hand back."

He modified his apparatus, so as to show by an index whether the pressure exerted was downwards, or in a horizontal direction. Where he parties thought they were pressing downwards, the index proved that they were pressing horizontally.

"When shown that it was so, they were truly surprised; but when they lifted up their hands and immediately saw the index return to its normal position they were convinced. When they looked at the index and could see for themselves whether they were pressing truly downward or obliquely so as to produce a resultant in the right or left-handed direction, then such an effect never took place. Several tried, for a long while together, and with the best will in the world; but no motion, right or left, of the table, or hand, or anything else, occured."

The effect of this test apparatus was to correct the mind of the table-movers. When they saw the index, it remained stationary; when hidden from their view, it moved under the pressure of their hands—a curious example of self-deception. Prof. F. continues—

"As soon as the index is placed before the most earnest, and they perceive—as in my presence they have always done—that it tells truly whether they are pressing downward only or obliquely, then all effects of table-turning cease, even though the parties persevere, earnestly desiring motion, till they become weary and worn out. No prompting or checking of the hands is needed—*the power is gone*; and this only because the parties are made conscious of what they are really doing mechanically and so are unable unwittingly to deceive themselves."

Just as there are but few people, comparatively, who can be "mesmerized," so there is but here and there an individual capable of this self-deception, and consequently there are not many so called "*media* ." It is only those able to impose upon themselves who can become "table-turners."

By the use of this instrument, says Prof. F., the mind of the table mover is instructed.

"The involuntary or *quasi* involuntaary montion is checked in the commencement, and therefore never rises up to the degree needful to move the table, or even permanently the index itself. No one can suppose that looking at the index can in any way intefere with the transfer of electricity or any other power from the hand to the board under it, or to the table. If the board tends to move, it may do so; the index does not confine it; and if the table tends to move, there is no reason why it should not. If both were influenced by any power to move together they may do so—as they did indeed when the apparatus was tied, and the mind and muscles left unwatched and unchecked."

We have not a doubt in the world, that this is the true explanation of these movements, which have seemed so mysterious, and have come nigh running crazy whole communities. We have constantly referred them to *muscular contractions exerted unconsiciously* by the individual, the same that execute the "Spiritual Writings," which we noticed some time ago and accounted for upon this principle. The philosphy of the whole matter is given in the last edition of Carpenters Physiology, to which we would refer for a full exposition of the subject. Prof. Faraday owns in conclusion, that he "is a little ashamed of his communication," so the reason that "he thinks, in the present age and in the part of the country" for which he wrote, "it ought not to have been required." We are glad that he took the subject up, his authority being the highest in all questions relating to physical science; but at the same time, we have no idea that his solution of the mystery will be satisfactory to the masses, or that it will suddenly cure the delusion. It must have its run; but fortunately, the malady is self-limited.

